# Evaluating population-level interventions to reduce inappropriate antibiotic use in healthcare and community settings: A systematic review protocol

**DOI:** 10.1371/journal.pone.0300780

**Published:** 2024-03-18

**Authors:** Shishi Wu, Olivia Magwood, Quanfang Dong, Xiaolin Wei

**Affiliations:** 1 Dalla Lana School of Public Health, University of Toronto, Toronto, Ontario, Canada; 2 Bruyère Research Institute, Ottawa, Ontario, Canada; 3 Faculty of Health Sciences, Interdisciplinary School of Health Sciences, University of Ottawa, Ottawa, Ontario, Canada; 4 Institute of Health Policy, Management and Evaluation, Dalla Lana School of Public Health, University of Toronto, Toronto, Ontario, Canada; SPHMMC: St Paul’s Hospital Millennium Medical College, ETHIOPIA

## Abstract

**Background:**

Inappropriate antibiotic use contributes significantly to the global challenge of antimicrobial resistance. While government-initiated population-level interventions are fundamental in addressing this issue, their full potential remains to be explored. This systematic review aims to assess the effectiveness of such interventions in reducing inappropriate antibiotic use among antibiotic providers and users in healthcare and community settings.

**Methods:**

We will conduct a systematic literature search across multiple databases and grey literature sources. We will include studies which evaluate the effectiveness of population-level interventions to reduce inappropriate antibiotic use in healthcare and community settings in both high-income and low- and middle-income countries. This includes government-initiated measures targeting antibiotic use through education, restriction, incentivization, coercion, training, persuasion, context modification, behavior modeling, or barrier reduction. Two reviewers will independently perform screening to select eligible studies, followed by data extraction. The outcomes of interest are various measures of antibiotic prescription and consumption, such as Defined Daily Dose (DDD) or number of prescriptions per year. We anticipate including a broad range of study designs and outcome measures. Therefore, we will narratively synthesize results using the categories of the population-level policy interventions of the Behavior Change Wheel Framework. We will organize outcome data by economic contexts, target populations, and implementation settings.

**Discussion:**

This review will strengthen the evidence base for the use of population-level interventions to address inappropriate antibiotic use. Drawing lessons from global experiences, the findings will provide valuable guidance to health policymakers, public health authorities, and researchers on tailoring interventions to specific economic contexts, populations, and settings, thereby enhancing their capacity to drive substantial improvement in appropriate antibiotic use.

## Introduction

Antimicrobial resistance (AMR) is a major threat to both human and animal health, causing increasing mortality and a growing economic burden globally [1]. In 2019, it was estimated that 4.95 million deaths were associated with AMR, and AMR will lead to 10 million deaths worldwide by year 2050 if inappropriate use of antimicrobials are left unchecked [2,3]. AMR infections are more expensive to treat resulting in greater health system costs, and in 2018 it was estimated that treating AMR and its complications cost up to US$3.5 billion per year across 33 Organization for Economic Co-operation and Development (OECD) countries [4]. Inappropriate use of antimicrobials, mostly antibiotics, in humans and animals is the major driver of AMR, and in many countries, a large proportion of inappropriate antibiotic use occurs at primary care settings and in communities.

Although the majority of United Nation Member States have published national action plans in response to the call from the World Health Organization (WHO) for national responses to address AMR as part of a One Health approach, efforts made by most countries may not be sufficient to meet the goals set in the plans. Public awareness campaigns and releasing guidelines for antibiotic prescribing remain the most commonly used strategies led by governments for reducing antibiotic use globally [5]. For example, a review of AMR policy actions in Canada identified 52 active programs and policies between 2008 and 2018, most of which were disjointed series of small education-based programs to improve public awareness of AMR and guidelines released by the federal or provincial governments. However, evidence on the effectiveness of public awareness programs is still inconclusive due to the poor quality of evaluation studies [6], and there is no evidence that shows what program elements make an effective public awareness campaign [5]. Evidence at the global level showed that single component interventions, such as disseminating guidelines alone, have limited impact [7,8], as studies show that interventions that are multifaceted, based on an assessment of individual and contextual barriers to change, and responsive to local circumstances are more likely to be successful [9,10].

Population-level interventions are defined as interventions enacted by a government or government agency at the national or sub-national level that aim to change antimicrobial use through education, restriction, incentivization, coercion, training, persuasion, changing the physical or social context, modelling appropriate behavior, or reducing barriers to action [11]. Although population-level interventions are developed and implemented with the aim to bring wider and more dramatic change (such as regulations and fiscal policies), they are rarely implemented and their full potential has yet to be explored [11]. A systematic review that was conducted in 2019 mapped and described the full range of government-led population-level policy actions, but their effectiveness was not assessed because most of these policies were either evaluated once or twice or not assessed by rigorous methods, hence unable to draw a strong conclusion [11]. In the following year, a systematic review assessed the impact of national interventions on promoting responsible antibiotic use. However, the review focused on interventions that were implemented at national level, which left out interventions that are led by other levels of governments and implemented at subnational level but may also have a wide impact on the population [12]. Additionally, several recent reviews have evaluated the effectiveness of a single type of population-level intervention. For example, financial strategies targeting healthcare providers are associated with improvement in appropriate antibiotic use [13]; communication interventions, such as public awareness campaign and public health interventions using social media, have limited impact on improving consumers’ knowledge and changing their behaviors [14]. To date, no existing review has provided a comprehensive assessment of the effectiveness of population-level interventions. However, high-quality evidence on the effectiveness of population-level interventions and context-specific analysis of elements contributing to successful implementation of population-level interventions in both high-income countries (HICs) and low- and middle-income countries (LMICs) are urgently needed.

Therefore, we aim to conduct a systematic review at the global level to evaluate the effectiveness of population-level interventions in reducing inappropriate antibiotic use among antibiotic providers and users in healthcare and community settings.

## Methods

We reported this systematic review protocol in accordance with PRISMA-P (S1 Checklist). The protocol has been registered online on the PROSPERO (CRD42023471043).

### Search strategy

We will search five electronic databases from inception up to September 2023, including MEDLINE (Ovid), CINAHL (Ebsco), EMBASE (Ovid), Cochrane library, and Web of Science. The search strategy was developed for Medline and reviewed by a librarian prior to translation for other databases (S1 File). Additionally, we will conduct a focused grey literature search by scanning the websites of the following international organizations: WHO, the National Institute for Health and Care Excellence, Agency for Healthcare Research and Quality, OECD, the World Bank, the Bill and Melinda Gates Foundation, the Oxford Martin School, and Public Health Agency of Canada (PHAC). A key world search will be performed using Google and Google scholar, and only the first ten pages of the search results will be reviewed. Finally, the reference lists of systematic and scoping reviews of similar topics will be checked manually. All search results will be imported to Covidence, which is an online program that facilitates screening and data extraction.

### Inclusion and exclusion criteria

We developed the inclusion and exclusion criteria guided by the Population, Exposure, Comparator and Outcome (PECO) framework [15], as summarized in Table 1. Our review will examine population-level interventions with an aim to reduce inappropriate antibiotic use among either antibiotic providers or users in communities and all healthcare settings (including primary, secondary, and tertiary).

**Table 1:** Summary of inclusion and exclusion criteria.

Domains	Inclusion criteria	Exclusion criteria
**Population**	• Antibiotic prescribers (e.g., physicians, dentists, nurses, and pharmacists) • Antibiotic users (e.g., the general public and patients seeking healthcare)	• Veterinarians and farm owners • Animal health
**Intervention**	Population-level interventions that aim to reduce inappropriate antibiotic use among the target populations	Interventions that do not meet the definition of “population-level interventions” or those with different aims and objectives
**Outcomes**	• Antibiotic prescription among providers • Antibiotic consumption among users	-
**Effect measurement**	We will include all metrics identified in the eligible studies for measuring changes in antibiotic prescription or consumption volume. These include but not limited to: • Total number of antibiotics prescribed/dispensed • Number of antibiotic packages/year • Number of antibiotics items per 1,000 population • Defined Daily Dose (DDD)	-
**Context**	All healthcare settings (including primary, secondary, and tertiary health settings) and communities	-
**Study type**	• Randomized controlled trial • Non-randomized controlled trial • Time series design • Non-randomized controlled before-and-after design	Qualitative studies, editorials, commentaries, conference abstracts/posters, and literature reviews
**Languages**	Arabic, Chinese, English, French, Russian, and Spanish	
**Publication year**	No restrictions	

Specifically, we will include antibiotic prescribers, such as physicians, dentists, nurses, and pharmacists, as well as unqualified staff or vendors who dispense antibiotics in communities in many LMICs. We will also include antibiotic users in communities, including the general public and patients seeking healthcare. We will exclude studies focused on veterinarians and farm owners because antibiotics specific to animal use are out of the scope of this review. Any population-level intervention to reduce antibiotic use will be included. Population-level interventions are defined as interventions enacted by a government or government agency at the national or sub-national level that aim to change antimicrobial use through education, restriction, incentivization, coercion, training, persuasion, changing the physical or social context, modelling appropriate behavior, or reducing barriers to action [11]. Any interventions that do not meet this definition will be excluded. To be included, a study must clearly describe the intervention with adequate description of the study aim, target population, timing, and content. Furthermore, interventions that have been implemented in communities and all levels of healthcare will be within the scope of our interest, because some interventions, such as regulation, legislation and fiscal, may apply to all healthcare settings.

In terms of the primary outcomes, we will include all measurements for antibiotic prescription by providers and consumption by users identified in the studies serving as indicators for assessing the effectiveness of the interventions. These measurements may include, but not limited to, total number of antibiotics prescribed/dispensed, number of antibiotic packages sold per year, or number of antibiotic items consumed per 1,000 population. Furthermore, we will include studies that assess the effectiveness of population-level interventions using the following additional outcomes (but not limited to): patient adherence to prescribed antibiotics, development of antibiotic resistance (resistance rates), patient and/or provider knowledge, attitudes, or beliefs about antibiotic use, compliance with national prescribing indicators.

Since our goal is to evaluate the effectiveness of population-level interventions on antibiotic use, we will include experimental designs (e.g., randomized controlled) and quasi-experimental designs (e.g., interrupted time-series analyses and before-and-after studies). Qualitative studies, editorials, commentaries, conference abstracts/posters, and literature reviews will be excluded. Studies conducted in HICs and LMICs are eligible. We will include articles published in any of the six official United Nations languages: Arabic, Chinese, English, French, Russian and Spanish.

### Screening

A two-stage screening process will be conducted to identify eligible studies. In the first stage of screening, two reviewers will independently review titles and abstracts of the retrieved studies. Full-text reports for all potentially relevant studies and for studies without an available abstract will be sent to the second stage of screening involving full-text review. Two reviewers will independently examine the full texts of the studies and exclude those that do not meet the eligibility criteria. Any disagreements during the screening process will be resolved by a third reviewer. Studies for which full-text reports cannot be retrieved through online databases and library searches will be excluded. The study selection process will be reported using a PRISMA flow diagram, including reasons for excluding full-text studies. The screening will be performed using COVIDENCE.

### Data extraction

A predesigned data extraction form will be developed for data extraction. Data extraction of the first ten articles will be done by both reviewers in duplicate. Any disagreement will be discussed and resolved via consensus or consulting a third reviewer. Once consistency in data extraction is achieved, extraction of the remaining articles will be split between the two reviewers. The lead author will be responsible for verifying the accuracy of all extracted data. Table 2 shows the data to be extracted from included studies.

**Table 2:** Data to be extracted from included studies.

Article information	Authors, title, publication year, study location
Study design	Study aim, study design, data collection methods, framework used (if any), sample size, description of study participants, analysis methods
Description of the intervention	Contextual information of the intervention, year of intervention conducted, implementation scale, targeted population(s) [antibiotic providers, antibiotic users], components of intervention
Description of assessment outcomes and results	Outcome measurements, key results on outcomes, study limitations, study conclusions, recommendations for policy, practice, or research

### Quality assessment

The quality of included studies will be assessed using the Cochrane Risk of Bias Tool (version 2) for randomized studies, and the Newcastle-Ottawa Scale (NOS) for nonrandomized studies [16,17]. Studies will be assessed in terms of selection bias, performance bias, detection bias, attrition bias, reporting bias, and other sources of bias, and a summary table will be produced. Two reviewers will carry out the assessment independently. The results of the assessment will be compared between the two reviewers, and any disagreement will be resolved by a third reviewer.

### Data synthesis

We will first tabulate and conduct a narrative synthesis of the characteristics of included studies. Subsequently, interventions that are described in these studies will be categorized based on the seven categories defined by the Behavioral Change Wheel Framework [18]. Table 3 summarizes the definition of the seven categories. Population-level interventions that we identify are likely to be implemented in different settings with specific goals and measured by related outcome indicators. We will also note studies that highlight interventions tailored to specific groups (e.g., based on gender, ethnicity, race, language). Given this diversity, we will narratively synthesize results on measured outcomes by each intervention category according to the Synthesis without meta-analysis (SWiM) in systematic reviews reporting guideline [19].

**Table 3:** Definitions of the seven intervention categories.

Intervention category	Definition
Communication	Using print, electronic, telephonic or broadcast/social media
Guideline	Creating documents that recommend or mandate clinical practice. This includes all changes to service provision
Fiscal	Using the tax system to reduce or increase the financial cost
Regulation	Establishing rules or principles of behavior or practice
Legislation	Making or changing laws
Environmental/social planning	Designing and/or controlling the physical or social environment
Service provision	Delivering a service

To further enhance the depth our analysis, we will stratify the evidence by several key factors. First, we will synthesize the evidence by HICs and LMICs to highlight the specific contexts for these interventions. This ensures that our analysis takes into account the different economic landscape in which the interventions were implemented, thus facilitating our understanding of the challenges and opportunities faced in various regions. Second, we will stratify the evidence by two population groups: antibiotic providers and users. This differentiation will help us better understand how population-level interventions impact distinct target populations. Finally, we will consider the settings in which the interventions were implemented. Specifically, we will perform subgroup synthesis based on their implementation settings, including primary care settings, community settings, hospitals, or other healthcare facilities, as we recognize that the settings can significantly influence the feasibility and impact of population-level interventions.

We will ensure our analysis includes sex, gender, and equity lens and describe in what ways interventions are currently shaped by these factors, as well as any gaps in sex, gender, and equity considerations in current interventions.

### Patient and public involvement

No patients or members of the public were involved in the development of this protocol.

## Discussion

This systematic review aims to comprehensively synthesize evidence on the effectiveness of population-level interventions in reducing inappropriate antibiotic use in healthcare and community settings. We will systematically search for and synthesize evidence on the impact of these interventions on antibiotic providers and users within the available literature and reveal how different economic contexts and settings can influence the effectiveness of these interventions.

Given the current evidence gap, the findings of this review will strengthen the evidence base for the use of population-level interventions as a strategic approach to tackle inappropriate antibiotic use, thus contributing to achieving the goals set in the national action plans. By showing the impact of these interventions on antibiotic providers and users, as well as shedding light on how different settings (encompassing healthcare and community settings) can shape their effectiveness, this review will provide health policymakers, public health authorities, and researchers with valuable guidance on the development and implementation of these interventions tailored to specific target populations and settings, thus enhancing their capacity to drive substantial improvement in appropriate antibiotic use. Moreover, the inclusion of both HICs and LMICs in this review acknowledges the global nature of the AMR challenge. Health policymakers and researchers worldwide can draw lessons from the interventions that have been implemented in different economic contexts, adapting successful strategies to their own settings. This cross-context learning is vital for crafting effective interventions and policies in the future.

### Strengths and limitations

We will follow a rigorous systematic and transparent approach to literature search, screening, data extraction, and evidence synthesis. Additionally, to ensure the comprehensiveness of our literature search, our search strategy was developed in consultation with a librarian and the searches will be performed encompassing multiple databases and grey literature sources. The inclusion of studies in six official United Nations languages will broaden the scope and inclusivity of the review.

This review has the following limitations. First, despite extensive efforts, there remains the possibility of missing some relevant studies, particularly those unpublished, solely available in the form of conference abstracts, or those not indexed in the databases included in our search. Second, the heterogeneity of interventions, settings, and outcomes used to evaluate the effectiveness of the interventions across studies may pose challenges in data synthesis and comparison. Finally, the quality of individual studies can vary widely. Low-quality studies can introduce bias and reduce the reliability of the review’s conclusions. However, if only few studies are available on a particular category of intervention or study setting, excluding lower-quality studies entirely could result in a lack of data or a skewed representation of the evidence. In such case, we will include studies with lower quality, while acknowledging their limitations. We believe that this approach will provide a more comprehensive overview of the available evidence.

## Supporting information

S1 Checklist PRISMA checklist. (DOCX)

S1 File Search strategy. (DOCX)
