# Supplementary material for: Evaluating population-level interventions to reduce inappropriate antibiotic use in healthcare and community settings: A systematic review protocol
Source: PLoS One. 2024 Mar 18;19(3):e0300780. doi: 10.1371/journal.pone.0300780 (PMC10947694; doi:10.1371/journal.pone.0300780)
Supplement: S1 File — (DOCX) [file pone.0300780.s002.docx]

**Supporting information S2. Search strategy used in OVID Medline**

| **Line #** | **Concept** | **Search terms** | **Hits** |
| --- | --- | --- | --- |
| 1 | AMR | exp Drug Resistance, Microbial/ | 402850 |
| 2 |  | (anti-bacterial* or antibacterial* or anti-mycobacterial* or antimycobacterial* or antibiotic* or antimicrobial* or anti-infective or antimicrobial agent* or anti-microbial agent* or antimicrobial resistance or anti-microbial resistance or antibiotic resistance or AMR).ti,ab,kf. | 664590 |
| 3 | Drug use/inappropriate use | drug prescription/ or drug utilization/ or exp inappropriate prescribing/ or exp drug misuse/ | 68498 |
| 4 |  | (stewardship or misuse or consume or consumption or prescription* or prescrib* or overprescrib* or sale* or rate* or "appropriate adj2 use" or "appropriate adj2 utiliz*" or "rational adj2 use" or "inappropriate adj2 use" or "inappropriate adj2 utiliz*" or "inappropriate adj2 prescrib*" or "drug misuse").ti,ab. | 4069980 |
| 5 | Interventions | exp health policy/ or policy making/ or legislation as Topic/ or Government Regulation/ or health plan implementation/ or Antimicrobial Stewardship/ or Legislation, Drug/ | 183730 |
| 6 |  | (program* or campaign* or policy or policies or guideline* or ban or banned or regulat* or law or laws or prohibit* or restrict* or legislat* or tax* or audit* or formular* or expenditure* or spending or label* or market* or advertis* or consultation*).ti,ab. | 5645728 |
| 7 | Study design | program evaluation/ or cost-effectiveness/ or interrupted time series analysis/ or controlled before-after studies/ or clinical trials as topic/ | 271320 |
| 8 |  | (randomized controlled trial or controlled clinical trial or pragmatic clinical trial).pt. | 691601 |
| 9 |  | (randomised or randomized or randomly or trial or evaluation).ti,ab. | 2809773 |
| 10 |  | ((before adj2 after adj5 (design$ or study or trial)) or (interrupt$ adj2 time series)).tw,kf. | 19569 |
| 11 |  | ((preintervention? or pre intervention? or postintervention? or post intervention?) adj5 (study or trial)).tw,kf. | 2863 |
| 12 |  | (pre adj2 post adj2 (design$ or method$ or study or trial)).tw,kf. | 10487 |
| 13 |  | ((quasi experimental or quasiexperimental or quasi randomi$ or quasirandomi$ or before-after) adj2 (design$ or method$ or study or trial)).tw,kf. | 18476 |
| 14 | Combined | (1 or 2) and (3 or 4) and (5 or 6) and (7 or 8 or 9 or 10 or 11 or 12 or 13) | 6914 |
| 15 | Population | exp animals/ not humans/ | 5155440 |
| 16 | Remove animal studies | 14 not 15 | 6685 |
